# Supplementary material for: Taste-Active Peptides from Triple-Enzymatically Hydrolyzed Straw Mushroom Proteins Enhance Salty Taste: An Elucidation of Their Effect on the T1R1/T1R3 Taste Receptor via Molecular Docking
Source: Foods. 2024 Mar 25;13(7):995. doi: 10.3390/foods13070995 (PMC11011393; doi:10.3390/foods13070995)
Supplement: Supplementary file 1 [file foods-13-00995-s001.zip › foods-2909602-supplementary.pdf]

Table S1 Free amino acid content of straw mushroom enzymatic digest

Amino acids	Content（mg/ml）		
	**Monoenzyme**	**Dual Enzyme**	**Trienzyme**
Asp	0.0862±0.01[b]	0.0856±0.02[b]	0.1091±0.01[a]
Glu	0.4301±0.02[c]	0.4412±0.01[b]	0.5425±0.03[a]
Ser	0.0310±0.01[b]	0.0290±0.02[c]	0.0422±0.01[a]
His	0.0467±0.01[a]	0.0412±0.01[a]	0.0550±0.01[b]
Gly	0.0503±0.01[c]	0.0547±0.01[b]	0.0604±0.01[a]
Thr	0.1695±0.01[b]	0.1567±0.01[b]	0.1871±0.01[a]
Arg	0.2386±0.02[b]	0.2113±0.01[b]	0.2574±0.01[a]
Ala	0.2208±0.01[a]	0.2124±0.01[a]	0.2590±0.01[a]
Tyr	0.1561±0.01[b]	0.1401±0.01[c]	0.1580±0.01[a]
Cys	0.0084±0.01[a]	0.0020±0.01[b]	0.0151±0.01[a]
Val	0.1494±0.01[a]	0.1373±0.01[c]	0.1624±0.01[b]
Met	0.0449±0.01[a]	0.0177±0.02[b]	0.0295±0.01[b]
Phe	0.2013±0.01[a]	0.1523±0.01[b]	0.1777±0.01[a]
Ile	0.1624±0.02[a]	0.1410±0.01[a]	0.1688±0.02[a]
Leu	0.2887±0.03[a]	0.1778±0.01[b]	0.2106±0.01[a]
Lys	0.2185±0.01[b]	0.1992±0.01[b]	0.2393±0.01[a]
Pro	0.0498±0.01[b]	0.0568±0.01[b]	0.0635±0.01[a]
Total	2.5526±0.02[b]	2.2563±0.06[c]	2.7377±0.05[a]

Note: Using a one-way ANOVA, the same letter of the marker indicates that there is no statistical difference between the amounts of free amino acids obtained using different enzymatic methods ($p>0.05$).

Table S2 Results of the peptide molecular weight distribution

Peptide molecular mass/Da	Mass fraction /%		
	**Monoenzyme**	**Dual Enzyme**	**Trienzyme**
<500	85.70±0.02[c]	86.33±0.03[b]	87.01±0.02[a]
500-1000	8.77±0.02[a]	8.54±0.01[b]	8.69±0.02[c]
1000-3000	4.33±0.01[c]	4.08±0.01[b]	3.31±0.02[a]
>3000	1.19±0.01[c]	1.06±0.01[b]	0.99±0.02[a]

Note: Using one-way ANOVA, the different letters of the markers indicate statistical differences in the content of peptides of different molecular weights obtained using different enzymatic digestion methods ($p>0.05$).

Table S3 The parameters of the power functions and the relationship between the natural logarithmic salt concentration and the natural logarithmic intensity of saltiness.

Control	A1	A2	A3
$y=1.21483x+0.682$	$y=1.0736x+1.06555$	$y=0.9207x+1.47065$	$y=1.04752x+1.15109$
($R^2=0.95546$)	($R^2=0.97125$)	($R^2=0.98241$)	($R^2=0.97629$)

Table S4 The dose-response relationship of three synthetic peptides on saltiness intensity of 0.5g/L NaCl solution

VP10+0.5g/L NaCl		YN9+0.5g/L NaCl		DF8+0.5g/L NaCl	
VP10 (mM)	saltiness intensity	YN9 (mM)	saltiness intensity	DF8 (mM)	saltiness intensity
0	1	0	1	0	1
0.2	2.0±0.1[b]	0.2	1.5±0.1[a]	0.2	7.5±0.3[b]
0.4	4.5±0.1[b]	0.4	2.5±0.3[a]	0.4	8.0±0.4[c]
0.8	6.5±0.3[b]	0.8	5.0±0.1[a]	0.8	8.5±0.6[c]
1.6	7.8±0.4[c]	1.6	6.5±0.1[a]	1.6	7.0±0.4[b]
3.2	8.0±0.2[c]	3.2	1.2±0.3[a]	3.2	2.5±0.5[b]

Note: Using one-way ANOVA, the different letters of the markers indicate statistical differences in the content of peptides of different molecular weights obtained using different enzymatic digestion methods ($p>0.05$).

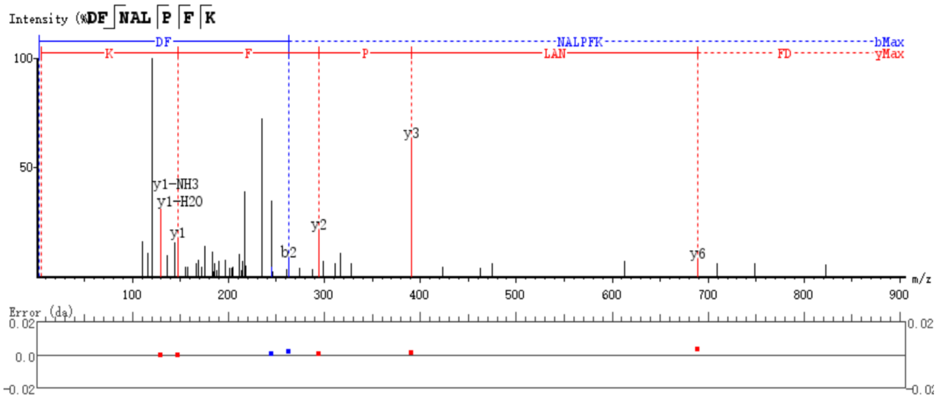
Intensity (%)
DF NAL P F K
DF
NALPFK
bMax
K
F
P
LAN
FD
yMax
y3
y1-NH3
y1-H2O
y1
y2
b2
y6
m/z
Error (da)

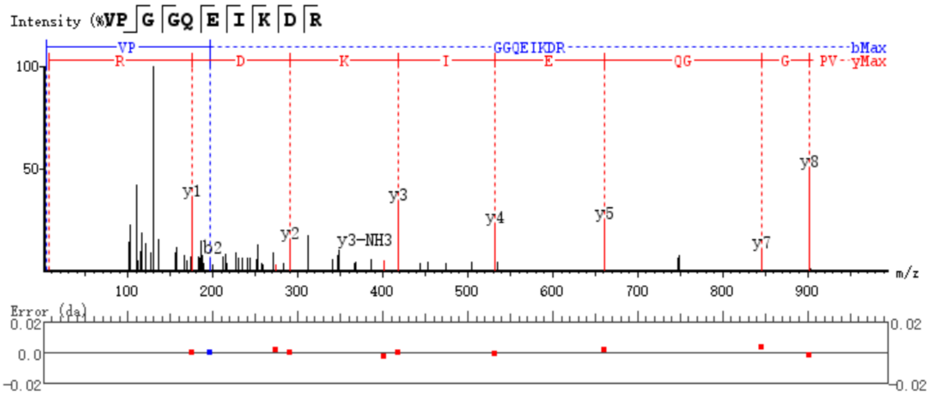
Intensity (%)
VP G GQ E I K D R
VP
GGQEIKDR
bMax
R
D
K
I
E
QG
G
PV
yMax
y8
y1
y3
y4
y5
y2
y3-NH3
b2
y7
m/z
Error (da)

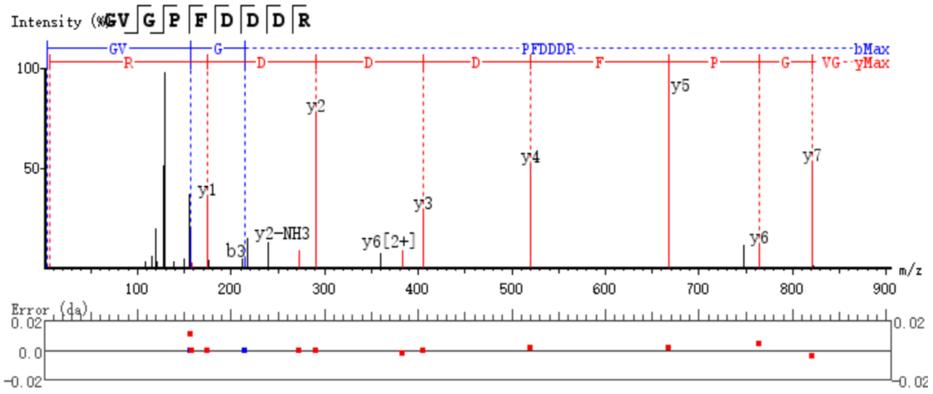
Intensity (%)
GV G P F D D D R
GV
G
PFDDDR
bMax
R
D
D
D
F
P
G
VG
yMax
y5
y2
y4
y7
y1
y3
y2-NH3
b3
y6[2+]
y6
m/z
Error (da)

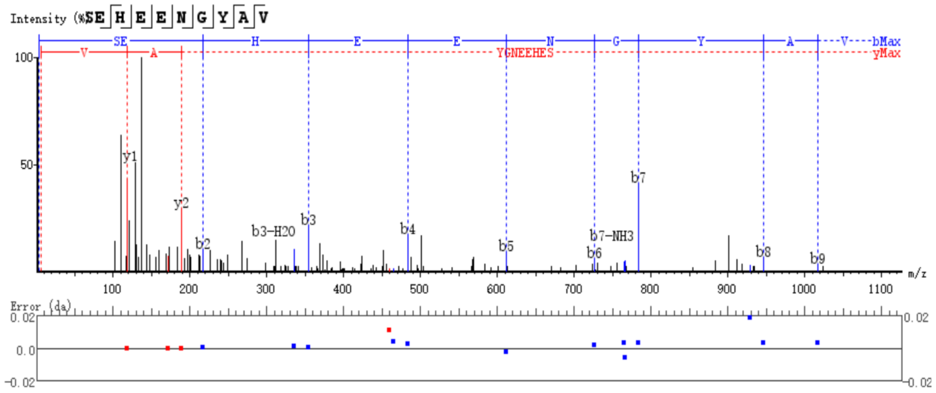
Intensity (%)
SE H E E N G Y A V
SE
H
E
E
N
G
Y
A
V
bMax
V
A
YGNEEHES
yMax
y1
b7
y2
b3-H2O
b3
b4
b2
b5
b7-NH3
b6
b8
b9
m/z
Error (da)

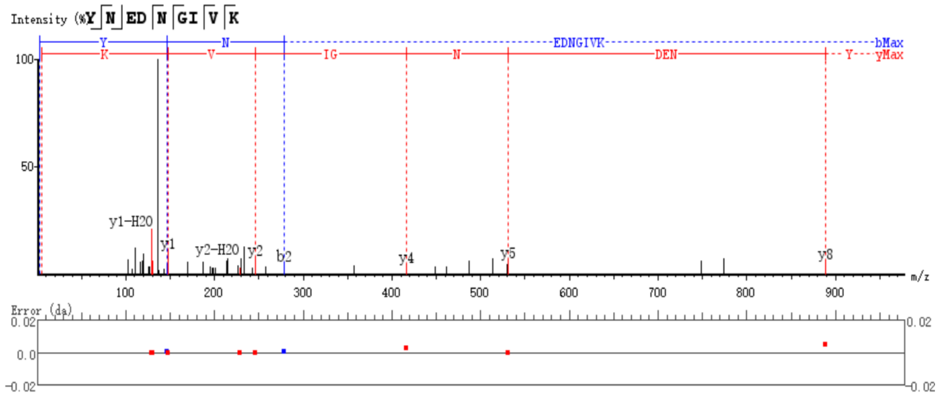
Intensity (%) Y N ED N GI V K
bMax
yMax
y1-H2O
y1
y2-H2O
y2
b2
y4
y5
y8
m/z
Error (da)

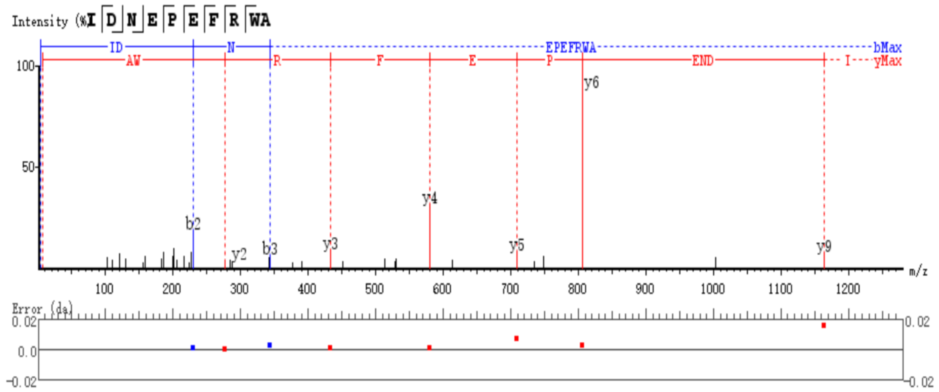
Intensity (%) I D N E P E F R WA
bMax
yMax
y6
y4
b2
y2
b3
y3
y5
y9
m/z
Error (da)

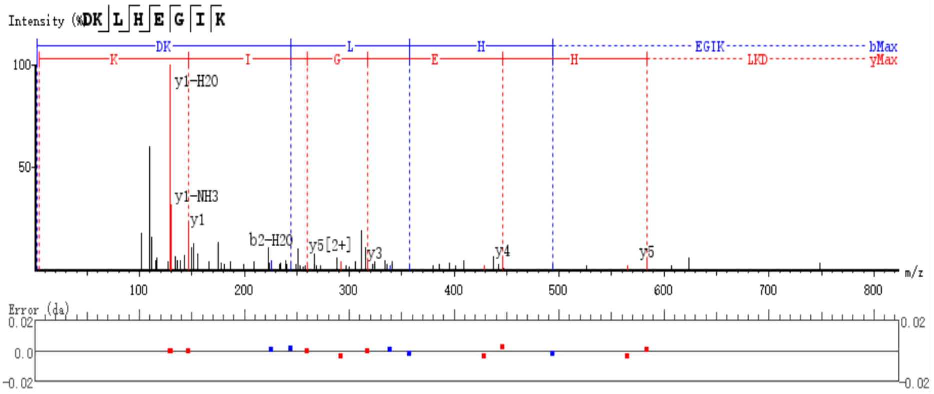
Intensity (%) DK L H E G I K
bMax
yMax
y1-H2O
y1-NH3
y1
b2-H2O
y5[2+]
y3
y4
y5
m/z
Error (da)

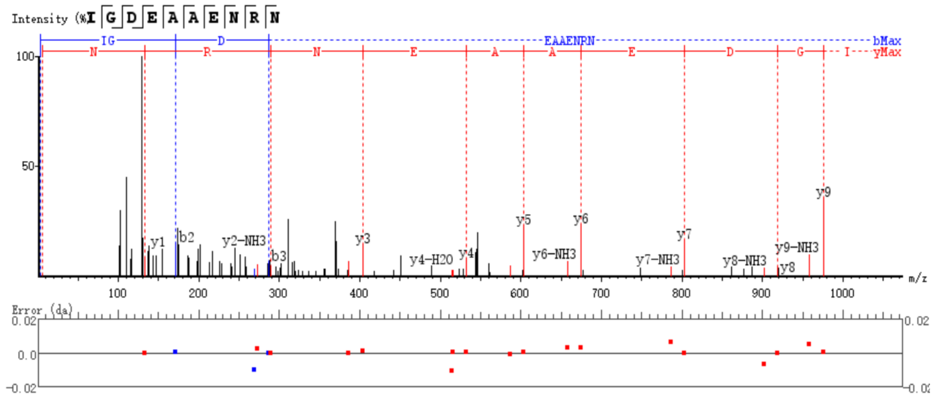
Intensity (%) I G D E A A E N R N
bMax
yMax
y9
y5
y6
y7
y1
b2
y2-NH3
b3
y3
y4-H2O
y4
y6-NH3
y7-NH3
y8-NH3
y9-NH3
y8
m/z
Error (da)

Figure S1. MS/MS spectra of DFNALPFK (m/z 574.25, A), VPGGQEIKDR (m/z 366.87, B), GVGPFDDDR (m/z 489.22, C), SEHEENGYAV (m/z 567.75, D), YNEDNGIVK (m/z 526.26, E), IDNEPEFRWA (m/z 638.81, F), DKLHEGIK (m/z 313.85, G), and IGDEAAENRN (m/z 544.75, H). The y and b ions refer to fragment ions of each peptide.
